# Domestic Correspondence

**Published:** 1884-01

**Authors:** D. B. Fonda

**Affiliations:** Jefferson


### Domestic Correspondence.

ARTICLE XI.

JEFFERSON, November 28, 1883.

TO THE CHICAGO MEDICAL JOURNAL AND EXAMINER:

In the November number of the JOURNAL appears an original communication from the pen of "S. V. Clevenger, M.D., Special Pathologist Cook County Insane Asylum," in which the writer treats of the early medical progress of the asylum, and in a manner which calls for more than a passing notice in the interest of justice, the profession, and the history of an institution now become so prominent and the source of such an extended public pride. The obligation to make such mention devolves upon me equally, perhaps, with any of the other individuals made mention of therein.

So far as space permits, I intend to give "an exact history" of the subject matter, as avowed to be the purpose of Dr. C. Unlike him, however, I am not prepared to admit, by any means, that the "paucity of asylum papers dating earlier than 1878" can interpose to baffle me in my humble efforts.

The great Chicago fire, while it has enabled the designing to cast clouds upon our titles to property in the destruction of valuable records relating thereto, has not destroyed living witnesses and private and public records of institutions to such an extent as to jeopardize the compilation of a fair history of any institution of prominence. Such a compilation is beyond the limits of space properly allowed to an article in the nature of a rejoinder, but it is among the contributions to be expected in the near future, and the ashes of the Chicago fire will not have to be disturbed in search for any of the materials requisite. It is sufficient at present to make not a simple correction but to give foundation to a protest in justice not only to myself, but to many, if not all of the officers connected with the county institutions in the earlier days, and when the same were laboring under embarassments now so happily removed. The pioneers that break the ground are not disposed to suffer a denial to some reasonable claims to a share in the fruitage.

It appears from the article in question that "from October 1, 1867, to January 10, 1871, Dr. D. B. Fonda had charge of the Poorhouse as medical visitor." This item of history is absolutely correct. Under the regime covering this period, it is vouchsafed that "the warden was a more important personage than the doctor;" but the compliment to the warden is nettled by the following:

"The welfare of a patient was insignificant alongside the chances of stealing a dollar or so in those days." Such a statement, read before a gathering of no less dignity than a medical society, would be impressive, if true. Having no foundation whatever, it bears a degree of ghoulish personality, not usually found in standard historical literature. I respectfully supplement the innuendo with the simple statement, that from the records still extant, it appears that during the said regime the cost of caring for the inmates of the institution reached the quarterly sum of only $17 per capita, a lower rate than appears at any time before or since said administration, and it does not appear from observation or from the records that the patients and inmates of the institution at that time fared less sumptuously than those of an earlier or subsequent date.

It appears further on in said article that "Dr. John W. Tope was appointed Medical Superintendent January 1, 1871." "*By this time* the poorhouse had outgrown itself, and the necessity for differentiating insanity from pauperism had become apparent enough to justify the erection of a large brick building capable of accommodating 300 patients." This statement furnishes an index by which to judge concerning many others contained in the article, but I will content myself with an allusion to it alone, not that it is a matter of supreme importance to the public to know what particular official has been instrumental,by council or report, in the establishment of an asylum for the insane separate from an institution for paupers, bat as giving the true history of this special movement, and in relief of the old corps of officers from the inuendo of having made a dollar in the public coffer of one department balance with the welfare of a patient in another department.

A reference to the official proceedings of the Board of Supervisors, held in December, A. D. 1876, discloses the correct historical data covering this subject. In the same report as then published, it appears that a report was presented to the Board by me as Physician for the County Poor House and Insane Asylum, specially setting forth the inadequacy of the one building occupied to meet the demands made upon the county by both the poor and the insane, and recommending that special provision be made for the accommodation of the insane, and that a separate building be erected for their exclusive occupancy, under a system of "differentiating" the two classes of public wards.

It appears further, that the Board took action on such report, and immediate steps were taken toward the erection of the present asylum building. The building was rapidly completed, and made ready for occupancy, and on January 1, 1871, the insane were transferred from the old quarters to the new, before my official career was ended, and under my personal superintendence as physician in charge.

Such is history, and in such brief terms its assertion. It is a subject of general congratulation now that the county's efforts to found a system broad enough to meet all public and charitable requirements, has resulted in a natural growth, and has reached so flattering a result, in the institutions now so admirably completed and systematized.

A fair history of their growth and a true appreciation of the humble efforts of the many who have labored to bring about the result, are not,I submit,enhanced by an article of so much prominence as the one referred to, and one teeming with so much reckless inaccuracy, and tinctured with such inexcusable and flagrant injustice. As will be noticed, I have not found it necessary for the purpose of this communication to refer to numerous authorities; but may be permitted to remark that the available resources of the records are not easily exhausted.

Concerning the "statistical information" given in tabular form, and forming the latter part of the article referred to, I have nothing to say as of serious interest to the public, since, if there be not a much larger degree of accuracy underlying the same than appears to have been displayed in the historical portion, the figures as tabulated lose claim to any degree of importance.

D. B. FONDA.

---

PROPHYLAXIS OF MALARIA.

The *Journal d'Hygiéne* has reproduced a report of Tommasi Crudeli to the Italian Parliament, on the prevention of malaria. He insisted strongly on the value of arsenic as a prophylactic, taken in a daily dose of 2 to 8 milligr.; he also mentioned the method advocated by Dr. Maglieri, of treating obstinate malarial infections by the administration of a simple decoction of lemons. This is the mode of preparation: Take a fresh lemon, and cut it in thin slices without peeling it; boil these slices in a new earthen pot with three glasses of water, until reduced down to a glassful; strain the whole through a cloth, and squeeze well the boiled lemon, and let the whole stand for a night to cool. Maglieri has tried this singular remedy not only in cases of chronic malarial infection, with a marked cachexia and rebelliousness to any other known remedy, but also in some cases of pernicious fevers, and always with success. The observations of Titus Piacentini confirm the efficacy of the decoction of lemons. Among the non-volatile substances so far found in the lemon, there are two, the *Hespéridine* ($C^{22}$, $H^{26}$, $O^{12}$), and the *Limonine* ($C^{26}$, $H^{30}$, $O^{5}$), which may be the active principles of this medicament. The former is found in all the parts of the fruit, the latter in the seed. But nothing certain is known of the physiological action of *hespéridine* and of *limonine*, and all the hypothesis that can be framed regarding this will be, in our present state of knowledge, entirely premature.—*Journal de Médecine et Chirurgie.*

